# Health and economic benefits of meeting WHO air quality guidelines, Western Pacific Region

**DOI:** 10.2471/BLT.22.288938

**Published:** 2022-12-08

**Authors:** Nicole Egerstrom, David Rojas-Rueda, Marco Martuzzi, Bin Jalaludin, Mark Nieuwenhuijsen, Rina So, Youn-Hee Lim, Steffen Loft, Zorana Jovanovic Andersen, Thomas Cole-Hunter

**Affiliations:** a Institute for Global Health (ISGlobal), Barcelona, Spain.; b Department of Environmental and Radiological Health Sciences, Colorado State University, Fort Collins, United States of America.; c Environment and Health Department, National Institute of Health, Rome, Italy.; d School of Population Health, University of New South Wales, Sydney, Australia.; e Section of Environmental Health, Department of Public Health, University of Copenhagen, Øster Farimagsgade 5, København K 1353, Denmark.

## Abstract

**Objective:**

To quantify the number of avoidable annual deaths and associated economic benefits from meeting the World Health Organization (WHO) air quality guidelines for ambient concentrations for fine particulate matter (PM_2.5_) for Member States of the WHO Western Pacific Region.

**Methods:**

Using the AirQ+ software, we performed a quantitative health impact assessment comparing country-level PM_2.5_ concentrations with the 2005 and 2021 air quality guidelines recommended maximum concentrations of 10 and 5 μg/m^3^, respectively. We obtained PM_2.5_ data from the WHO Global Health Observatory (latest available year 2016), and population and mortality estimates from the United Nations World Population Prospects database for the latest 5-year period available (2015–2019), which we averaged to 1-year estimates. A risk estimate for all-cause mortality, based on a meta-analysis, was embedded within AirQ+ software. Our economic assessment used World Bank value of a statistical life adjusted to country-specific gross domestic product (latest available year 2014).

**Findings:**

Data were complete for 21 of 27 Member States. If these countries achieved the 2021 guidelines for PM_2.5_, an estimated 3.1 million deaths would be avoided annually, which are 0.4 million more deaths avoided than meeting the 2005 guidelines. China would avoid the most deaths per 100 000 population (303 deaths) and Brunei Darussalam the least (5 deaths). The annual economic benefit per capita ranged from 5781 United States dollars (US$) in Singapore to US$ 143 in Solomon Islands.

**Conclusion:**

Implementing effective measures to reduce PM_2.5_ emissions would save a substantial number of lives and money across the Region.

## Résumé

**Objectif:**

Déterminer le nombre de décès annuels évitables et les avantages économiques associés au respect des lignes directrices de l'Organisation mondiale de la Santé (OMS) relatives à la qualité de l'air, notamment vis-à-vis des concentrations de particules fines (PM_2,5_) dans l'air ambiant, au sein des États Membres de la Région du Pacifique occidental de l'OMS.

**Méthodes:**

À l'aide du logiciel AirQ+, nous avons procédé à une évaluation quantitative de l'impact sur la santé en comparant les concentrations de particules PM_2,5_ à l'échelle nationale avec les lignes directrices de 2005 et 2021 relatives à la qualité de l'air, qui préconisent des concentrations de 10 et 5 µg/m^3^ respectivement. Nous nous sommes procuré des données sur les particules PM_2,5_ auprès de l'Observatoire mondial de la santé de l'OMS (la version disponible la plus récente datant de 2016). Les estimations concernant la population et la mortalité proviennent quant à elles de la base de données des Nations Unies, Perspectives de la population mondiale, pour la dernière période de cinq ans disponible (2015–2019), que nous avons ensuite divisée en moyennes annuelles. Le logiciel AirQ+ comprenait une estimation des risques pour la mortalité toutes causes confondues, fondée sur une méta-analyse. Enfin, notre évaluation économique reposait sur les valeurs de la vie statistique de la Banque mondiale, ajustées en fonction du produit intérieur brut de chaque pays (2014 étant l'année disponible la plus récente).

**Résultats:**

Les données se sont révélées complètes pour 21 des 27 États Membres. Si ces pays appliquaient les lignes directrices de 2021 pour les particules PM_2,5_, près de 3,1 millions de décès pourraient être évités chaque année, selon nos estimations. Ce qui représente 0,4 million de décès évitables supplémentaires par rapport aux chiffres escomptés pour les lignes directrices de 2005. C'est la Chine qui pourrait ainsi éviter le plus de décès par 100 000 habitants (303 morts), tandis que le Brunéi Darussalam en éviterait le moins (5 morts). L'avantage économique annuel par habitant était compris entre 5781 dollars américains (USD) à Singapour et 143 USD dans les Îles Salomon.

**Conclusion:**

Déployer des mesures efficaces visant à réduire les émissions de particules PM_2,5_ permettrait de sauver un nombre considérable de vies et de réaliser des économies dans la Région.

## Resumen

**Objetivo:**

Cuantificar el número de muertes anuales evitables y los beneficios económicos asociados al cumplimiento de las directrices sobre la calidad del aire de la Organización Mundial de la Salud (OMS) relativas a las concentraciones ambientales de partículas finas (PM_2,5_) para los Estados Miembros de la Región del Pacífico Occidental.

**Métodos:**

Utilizando el software AirQ+, se realizó una evaluación cuantitativa del impacto en la salud comparando las concentraciones de PM_2,5_ a nivel de país con las concentraciones máximas recomendadas por las directrices sobre la calidad del aire de 2005 y 2021 de 10 y 5 μg/m^3^, respectivamente. Se obtuvieron datos de PM_2,5_  del Observatorio Mundial de la Salud de la OMS (último año disponible 2016), y estimaciones de población y mortalidad de la base de datos World Population Prospects de las Naciones Unidas para el último periodo de 5 años disponible (2015-2019), que se promediaron a estimaciones de 1 año. El software AirQ+ incorporó una estimación del riesgo de mortalidad por todas las causas a partir de un metanálisis. En la evaluación económica, se utilizaron los valores estadísticos de vida del Banco Mundial ajustados al producto interior bruto específico de cada país (último año disponible: 2014).

**Resultados:**

Los datos estaban completos para 21 de los 27 Estados Miembros. Si estos países cumplieran las directrices de 2021 para las PM_2,5_, se evitarían unos 3,1 millones de muertes al año, lo que supone 0,4 millones de muertes más que si se cumplieran las directrices de 2005. China sería el país que evitaría más muertes por cada 100 000 habitantes (303 muertes) y Brunei Darussalam el que menos (5 muertes). El beneficio económico anual per cápita oscilaría entre los 5781 dólares estadounidenses (US$) de Singapur y los US$ 143 de las Islas Salomón.

**Conclusión:**

La adopción de medidas eficaces para reducir las emisiones de PM_2,5_ salvaría un número significativo de vidas y ahorraría dinero en toda la Región.

## ملخص

**الغرض:**

تحديد عدد حالات الوفاة السنوية التي يمكن تجنبها، والفوائد الاقتصادية المرتبطة بالوفاء بإرشادات جودة الهواء الصادرة عن منظمة الصحة العالمية (WHO) لتركيزات الجسيمات الدقيقة ( _2.5 _PM) للدول الأعضاء في منطقة غرب المحيط الهادئ التابع لمنظمة الصحة العالمية.

**الطريقة:**

باستخدام برنامج AirQ+‎، قمنا بإجراء تقييم كمي للأثر الصحي بمقارنة تركيزات _2.5 _PM على المستوى القطري، مع التركيزات القصوى التي تبلغ 10 و5 ميكروجرام/متر مكعب، والموصى بها في إرشادات جودة الهواء لعامي 2005 و2021 على التوالي. حصلنا على بيانات _2.5 _PM من مرصد الصحة العالمية التابع لمنظمة الصحة العالمية (أحدث عام متاح 2016)، وتقديرات السكان والوفيات من قاعدة بيانات التوقعات السكانية العالمية للأمم المتحدة لآخر فترة 5 سنوات متاحة (2015 إلى 2019)، والتي حسبنا متوسطها إلى تقديرات عام واحد. تم تضمين تقدير مخاطر الوفيات لجميع الأسباب، بناءً على التحليل التلوي، في برنامج AirQ+‎. استخدم تقييمنا الاقتصادي قيم البنك الدولي للحياة الإحصائية المعدلة وفقًا للناتج المحلي الإجمالي الخاص بكل دولة (آخر عام متاح 2014).

**النتائج:**

كانت البيانات كاملة بالنسبة لـ 21 دولة من أصل 27 دولة عضو. إذا تمكنت هذه الدول من تحقيق المبادئ التوجيهية لعام 2021 لـ _2.5 _PM ، فسيتم تجنب ما يقدر بنحو 3.1 مليون حالة وفاة سنويًا، وهو ما يزيد بمقدار 0.4 مليون حالة وفاة عن تلبية المبادئ التوجيهية لعام 2005. ستتجنب الصين أكبر عدد من حالات الوفاة لكل 100000 من السكان (303 حالة وفاة)، وستتجنب بروناي دار السلام أقل عدد من حالات الوفاة (5 وفيات). وتراوح نصيب الفرد من الفوائد الاقتصادية السنوية من 5781 دولارًا أمريكيًا في سنغافورة، و143 دولارًا أمريكيًا في جزر سليمان.

**الاستنتاج:**

إن تنفيذ تدابير فعالة للحد من انبعاثات _2.5 _PM من شأنه أن ينقذ عددًا كبيرًا من الأرواح، وقدرًا هائلًا من الأموال في جميع أنحاء المنطقة.

## 摘要

**目的:**

旨在确定世卫组织西太平洋地区成员国在实现世界卫生组织 (WHO) 空气质量指南中制定的细颗粒物 (PM_2.5_) 环境浓度达标后每年可避免的死亡人数和相关经济效益。

**方法:**

我们使用 AirQ+ 软件，开展了一项定量健康影响评估，比较了国家制定的 PM_2.5_ 浓度与 2005 年和 2021 年空气质量指南建议的最高浓度（分别为 10 和 5m克/立方米）。我们从世界卫生组织全球卫生观测站（最新可用数据年份 2016 年）获得了 PM_2.5_ 的数据，从联合国世界人口展望数据库获得了近 5 年（2015 - 2019 年）的人口和死亡率估计值，并计算出这些估计值的年平均值。AirQ+ 软件纳入了基于 meta 分析的全因死亡率风险评估。我们的经济评估使用了世界银行统计的按各国国内生产总值调整后的寿命值，（最新可用数据年份 2014 年）。

**结果:**

27 个成员国中有 21 个国家的数据是完整的。如果这些国家能够实现 2021 年指南中的 PM_2.5_ 浓度达标估计每年将避免 310 万人死亡，与实现 2005 年指南中的 PM_2.5_ 浓度达标相比，可多避免 40 万人死亡。每 10 万人中，中国将避免的死亡人数最多（303 人），文莱达鲁萨兰国可避免的死亡人数最少（5 人）。每年可带来的人均经济效益从 5781 美元（新加坡）到 143 美元（所罗门群岛）不等。

**结论:**

采取能有效减少 PM_2.5_ 排放的措施可以在整个西太平洋地区挽救许多生命并节省大量资金。

## Резюме

**Цель:**

Количественно определить число предотвратимых ежегодных смертей и связанные с этим экономические выгоды от соблюдения руководящих принципов Всемирной организации здравоохранения (ВОЗ) по обеспечению надлежащего качества воздуха в отношении концентрации в окружающей среде мелких твердых частиц (PM_2.5_) для государств-членов ВОЗ западной части Тихоокеанского региона.

**Методы:**

С использованием программного обеспечения AirQ+ была проведена количественная оценка воздействия на здоровье, в ходе которой концентрации PM_2.5_ на уровне страны сравнивались с рекомендованными в соответствии с руководящими принципами по обеспечению надлежащего качества воздуха на 2005 и 2021 годы максимальными концентрациями 10 и 5 мкг/м^3^ соответственно. Глобальная обсерватория здравоохранения ВОЗ позволяет получить данные по PM_2.5_ (последний доступный год – 2016 г.), а также оценки численности населения и смертности из базы данных ООН «Мировые демографические перспективы» за последний доступный 5-летний период (2015–2019 гг.), которые усреднялись до оценки за 1 год. В программное обеспечение AirQ+ была встроена оценка риска смертности по всем причинам, основанная на метаанализе. При проведении экономической оценки использовались показатели среднестатистической продолжительности жизни, скорректированные Всемирным банком с учетом валового внутреннего продукта конкретной страны (последний доступный год – 2014 г.).

**Результаты:**

Данные были полными по 21 из 27 государств-членов. При условии соблюдения этими странами руководящих принципов по PM_2.5_ в 2021 году ежегодно можно было бы предотвратить примерно 3,1 миллиона смертей, что на 0,4 миллиона случаев больше в сравнении с соблюдением руководящих принципов 2005 года. Китай избежал бы наибольшего числа смертей на 100 000 населения (303 смерти), а Бруней-Даруссалам – наименьшего  (5 смертей). Годовая экономическая выгода на душу населения варьировалась от 5781 доллара США (US$) в Сингапуре до 143 долларов США на Соломоновых островах.

**Вывод:**

Внедрение эффективных мер по снижению выбросов PM_2.5_ позволит спасти значительное количество жизней и сэкономить деньги по всему региону.

## Introduction

Air pollution is a major determinant of health and causes annually an estimated 6.7 million premature deaths worldwide. Only high blood pressure, tobacco use and dietary risks surpass air pollution as causing more premature deaths.^1^ Evidence suggests that fine particulate matter (particles ≤ 2.5 µm in diameter, PM_2.5_) is the main air pollution component causing harm, contributing annually to an estimated 4.1 million premature deaths worldwide.^2^ Accordingly, PM_2.5_ is one of the most monitored, regulated and studied air pollutants.^2^ Some of the highest ambient concentrations of PM_2.5_ are recorded in Member States within the World Health Organization (WHO) Western Pacific Region. While this Region only hosts one quarter of the world’s population, approximately one third of the global PM_2.5_-attributable deaths happen here.^1^

A systematic review of cohort studies conducted in the Region and a worldwide meta-analysis support the association between long-term exposure to PM_2.5_ and increased all-cause mortality.^3^^,^^4^ While PM_2.5_ concentrations are decreasing in some areas of the Western Pacific Region,^5^ the increases in major sources of PM_2.,5_, such as traffic, industry, energy production and agriculture, are putting populations at higher risk from ambient compared to indoor levels of PM_2.5_.^6^ Thus, assessing outdoor PM_2.5_ concentrations’ impact on public health is important to protect populations.

Some sources of PM_2.5_ are easier to manage than others. Localized sources may be addressed at the community or country levels, such as by implementing lower-emission transport policies: motorized traffic is a major contributor to localized PM_2.5_ levels in South-East Asian and Oceanian countries.^7^ However, transboundary air pollution is a major source of PM_2.5_ in countries such as Republic of Korea,^8^ with naturally-sourced dust a major contributor in northern China,^7^ requiring coordinated regional action with neighbouring countries.

To minimize harm, WHO has developed global air quality guidelines to support policy-makers. The guidelines focus on particulate matter, ozone, nitrogen dioxide and sulfur dioxide.^9^^,^^10^ In 2021, WHO updated the 2005 guidelines^10^ using evidence collected in the preceding 15 years. WHO realized that much more ambitious targets for ambient air pollution concentrations were needed to minimize harm,^9^ and in the updated guidelines the annual ambient concentration target for PM_2.5_ was halved from 10 to 5 µg/m^3^. This target is an ideal scenario that policy-makers may want to achieve through regulations.

Tools and measures are available for decision-makers to successfully manage air pollution through policy implementation and action. For example, a report from the United Nations (UN) Environment Programme provides 25 science-based clean air measures for policy-makers in Asia and the Pacific.^11^ The WHO-developed AirQ+ software supports health professionals and institutions to quantify the effects of ambient air pollution exposure through health impact assessments.^12^ Furthermore, policy-makers may consider the value of a statistical life, that is, an economic value used to quantify the benefit of avoiding a death, as a critical parameter in economic analyses for benefits and costs of regulatory actions.^13^

Here, we aimed to estimate the impact of Western Pacific Region’s Member States achieving the WHO air quality guidelines for PM_2.5_ annual ambient concentrations,^9^^,^^10^ by quantifying the number of avoided deaths and related economic benefit.

## Methods

### Data

Detailed description of data used in the health impact assessment is available in the online repository.^14^

We obtained the latest population-weighted country estimates of ambient PM_2.5_ concentrations (year 2016) from the indicator metadata registry list in WHO’s Global Health Observatory.^15^ We used combined total estimates for residence area types (rural and urban), as separate estimates were not available for many countries and the in-built AirQ+ functionality currently only accommodates country-wide estimates. As such, we assumed that all subregions within each country have the same concentrations for this specific year. 

For population and mortality data, we focused on individuals 30 years or older because concentration–response functions are limited to this higher age range; individuals younger than 30 years old are not expected to die from diseases caused by air pollution. We obtained 2015–2019 population and mortality estimates for each country from the UN World Population Prospects.^16^ A 1-year average of this 5-year period was calculated for all countries with available data; assuming that year-to-year fluctuations occur, thus an average providing a more accurate temporal estimate.

### Analyses

#### Concentration–response function

Our analysis used all-cause mortality attributable to air pollution as the outcome for ease of comparison across countries, in absolute and population-adjusted (per 100 000 population) terms. We calculated the estimated PM_2.5_-attributable proportional mortality as the estimated number of attributable cases divided by the population per 100 000 and the mortality rate per 100 000 people.

We performed a quantitative health impact assessment using AirQ+ version 2.1.1 (WHO European Region, Copenhagen, Denmark),^17^ which has in-built concentration–response functions derived from published scientific literature. In addition to the all-cause mortality concentration–response function, the software provides responses for mortality from specific causes, including lung cancer, chronic obstructive pulmonary disease, ischaemic heart disease and stroke, among adults. We decided to focus on mortality from all causes combined for simplicity. Within AirQ+ software, we used the embedded standard relative risk (RR) of the concentration–response function for all-cause mortality at a given level of outdoor PM_2.5_ concentration, that is RR: 1.08 (95% confidence interval, CI: 1.06–1.09). This RR is from a global meta-analysis of 104 studies.^4^

We adapted the above-listed data for input to AirQ+, and visualized AirQ+ health impact assessment results using R version 4.0.5 (R Foundation, Vienna, Austria)^18^ in RStudio version 1.4.1106 (Posit, Boston, United States of America),^19^ with packages *dplyr*, *ggplot2*, *readr*, *readxl* and *tidyr*.

As the factual for the health impact assessments, we used the annual estimates of country-level ambient PM_2.5_ concentration. As counterfactuals for the health impact assessments, we used the 2021 and 2005 annual ambient air quality guidelines PM_2.5_ target concentration values of 5 and 10 µg/m^3^, respectively. We used obtained population and mortality data to evaluate the standing of countries towards meeting the WHO air quality guidelines.

#### Economic assessment

To demonstrate the economic benefit of meeting the 2021 WHO air quality guidelines among the Western Pacific Region Member States, we used the value of a statistical life per Member State.^13^ Briefly, the value of a statistical life per Member State was calculated and published previously with adjustment by each country’s 2015 World Bank income classification for gross domestic product in 2014.^13^ The published values of a statistical life were calculated on a base value of a statistical life of 9.6 million United States dollars (US$) (in 2014), a sum which is based on United States labour market estimates. We calculated the absolute economic benefit per country as the estimated number of attributable deaths to PM_2.5_ concentrations above the air quality guidelines multiplied by the value of a statistical life per country in US$ millions. For easier interpretation and comparison across countries, we calculated values per capita per country by dividing the absolute economic value per country by population of that country. The value of a statistical life was not available for all countries, thus we only conducted an economic assessment for those countries with available data.

## Results

Estimates for mortality and population were available for 21 of 27 Member States; Cook Islands, Marshall Islands, Nauru, Niue, Palau and Tuvalu lacked data. Only Cook Islands and Niue had no available value of a statistical life data. We therefore excluded these six countries from our analysis; however, as small island states, we expect them to contribute with a very small percentage of the total regional values for population, deaths and economic benefit. We observed the highest annual concentrations of PM_2.5_ in a cluster of the northern Member States: China (49.16 µg/m^3^), Mongolia (40.42 µg/m^3^) and Viet Nam (29.66 µg/m^3^), while the lowest annual concentrations were seen in New Zealand (5.73 µg/m^3^), Brunei Darussalam (5.78 µg/m^3^) and Australia (7.19 µg/m^3^; Table 1).

**Table 1:** Description of data used in the health impact assessment of achieving WHO air quality guidelines, Western Pacific Region, 2015–2019

Country	Population 30 years or older, in millions^a^	All-cause mortality per 100 000 population^b^	Annual mean of PM_2.5_ concentration(µg/m^3^)^c^	Value of a statistical life, US$ millions^d^
Australia	14.71	1086	7.19	10.335
Brunei Darussalam	0.21	801	5.78	6.539
Cambodia	6.35	1192	23.98	0.184
China	852.00	1144	49.16	1.364
Cook Islands	NA	NA	12.03	NA
Fiji	0.40	1552	10.19	0.831
Japan	92.63	1422	11.45	6.682
Kiribati	0.04	1166	10.45	0.583
Lao People's Democratic Republic	2.54	1300	24.49	0.299
Malaysia	14.51	1023	16.04	1.819
Marshall Islands	NA	NA	9.43	0.821
Micronesia (Federated States of)	0.04	1430	10.23	0.612
Mongolia	1.36	1271	40.42	0.666
Nauru	NA	NA	12.53	2.653
New Zealand	2.76	1165	5.73	6.885
Niue	NA	NA	11.47	NA
Palau	NA	NA	12.18	2.095
Papua New Guinea	2.99	1485	10.91	0.385
Philippines	41.94	1193	18.38	0.611
Republic of Korea	34.25	869	24.57	4.723
Samoa	0.07	1229	10.56	0.676
Singapore	3.77	665	18.26	8.962
Solomon Islands	0.21	1012	10.67	0.330
Tonga	0.04	1700	10.08	0.736
Tuvalu	NA	NA	11.42	1.072
Vanuatu	0.10	1283	10.31	0.545
Viet Nam	48.29	1103	29.66	0.342

NA: not available; PM_2.5_: particulate matter with particles ≤ 2.5 µm in diameter; US$: United States dollars; WHO: World Health Organization.

^a^ Rounded-up to two decimal places.

^b^ In population 30 years or older.

^c^ Both rural and urban areas included; 2016 estimates.

^d^ Value of a statistical life per Member State is calculated by assuming a base value of a statistical life of US$ 9.6 million (in 2014), adjusted by a Member State’s income classification of 2015 from the World Bank.

### Health impact assessment

**Fig. 1:**
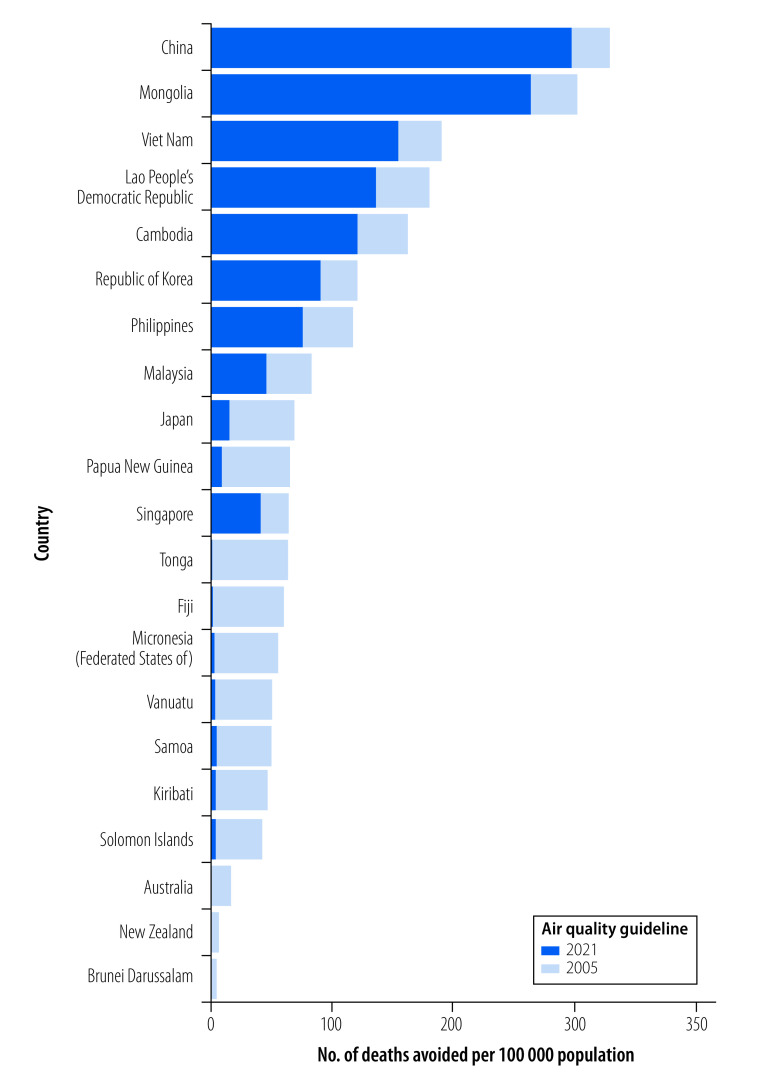
Annual deaths avoided per Member State of the WHO Western Pacific Region achieving 2005 or 2021 WHO air quality guidelines for annual PM_2.5_ concentration

The estimated number of attributable deaths from annual exposure to ambient PM_2.5_ concentrations above the 2005 air quality guidelines (10 µg/m^3^) or the 2021 air quality guidelines (5 µg/m^3^) is shown in Fig. 1 and Table 2. In the Western Pacific Region, an estimated 3 119 353 deaths would be avoided annually if the 2021 air quality guidelines for annual PM_2.5_ concentration was achieved, which is an additional 403 504 deaths avoided compared to achieving the 2005 air quality guidelines (Table 2).

PM_2.5_: particulate matter with particles ≤ 2.5 µm in diameter; WHO: World Health Organization.

**Table 2:** Estimates of avoidable deaths if Western Pacific Region Member States meet WHO targets for PM_2.5_ concentrations

Country	Attributable proportion, % (95% CI)^a^			No. of deaths (95% CI)			No. of deaths per 100 000 population at risk (95% CI)^b^	
	2005 guidelines	2021 guidelines		2005 guidelines	2021 guidelines		2005 guidelines	2021 guidelines
Australia	0.00 (0.00–0.00)	1.67 (1.27–1.87)		0 (0–0)	2 670 (2 026–2 987)		0 (0–0)	18 (14–20)
Brunei Darussalam	0.00 (0.00–0.00)	0.60 (0.45–0.67)		0 (0–0)	10 (8–11)		0 (0–0)	5 (4–5)
Cambodia	10.20 (7.82–11.35)	13.59 (10.47–15.09)		7 726 (5 925–8 596)	10 293 (7 930–11 428)		122 (93–135)	162 (125–180)
China	26.02 (20.40–28.64)	28.81 (22.69–31.65)		2 536 156 (1 988 549–2 791 767)	2 808 356 (2 211 321–3 085 088)		298 (233–328)	330 (260–362)
Fiji	0.15 (0.11–0.16)	3.92 (2.98–4.37)		9 (7–10)	241 (184–270)		2 (2–3)	61 (46–68)
Japan	1.11 (0.84–1.24)	4.84 (3.69–5.41)		14 617 (11 082–16 357)	63 790 (48 586–71 219)		16 (12–18)	69 (52–77)
Kiribati	0.35 (0.26–0.39)	4.11 (3.13–4.59)		2 (1–2)	20 (15–22)		4 (3–5)	48 (36–53)
Lao People's Democratic Republic	10.55 (8.10–11.74)	13.93 (10.74–15.46)		3 479 (2 670–3 871)	4 593 (3 540–5 098)		137 (105–153)	181 (140–201)
Malaysia	4.54 (3.46–5.07)	8.15 (6.23–9.08)		6 744 (5 134–7 530)	12 094 (9 250–13 474)		46 (35–52)	83 (64–93)
Micronesia (Federated States of)	0.18 (0.13–0.20)	3.95 (3.00–4.41)		1 (1–1)	24 (18–26)		3 (2–3)	56 (43–63)
Mongolia	20.87 (16.24–23.06)	23.86 (18.65–26.31)		3 605 (2 806–3 983)	4 121 (3 221–4 544)		265 (206–293)	303 (237–334)
New Zealand	0.00 (0.00–0.00)	0.56 (0.42–0.63)		0 (0–0)	180 (136–201)		0 (0–0)	7 (5–7)
Papua New Guinea	0.70 (0.53–0.78)	4.45 (3.39–4.97)		4 347 (3 294–4 865)	27 695 (21 084–30 928)		10 (8–12)	66 (50–74)
Philippines	6.25 (4.77–6.97)	9.78 (7.50–10.89)		31 252 (23 846–34 862)	48 962 (37 529–54 494)		75 (57–83)	117 (89–130)
Republic of Korea	10.61 (8.14–11.80)	13.98 (10.78–15.52)		31 567 (24 222–35 115)	41 609 (32 072–46 185)		92 (71–103)	122 (94–135)
Samoa	0.43 (0.33–0.48)	4.19 (3.19–4.68)		4 (3–4)	37 (28–41)		5 (4–6)	51 (39–57)
Singapore	6.16 (4.70–6.87)	9.70 (7.44–10.80)		1 543 (1 177–1 721)	2 430 (1 863–2 705)		41 (31–46)	65 (49–72)
Solomon Islands	0.51 (0.39–0.58)	4.27 (3.25–4.77)		11 (8–12)	89 (68–99)		5 (4–6)	43 (33–48)
Tonga	0.06 (0.05–0.07)	3.83 (2.92–4.28)		0 (0–0)	25 (19–28)		1 (1–1)	65 (50–73)
Vanuatu	0.24 (0.18–0.27)	4.00 (3.05–4.47)		3 (2–3)	49 (37–54)		3 (2–3)	51 (39–57)
Viet Nam	14.04 (10.82–15.59)	17.29 (13.38–19.15)		74 783 (57 647–83 004)	92 065 (71 284–101 965)		155 (119–172)	191 (148–211)
**Total**	**NA**	**NA**		**2 715 849 (2 126 374**–**2 991 703)**	**3 119 353 (2 450 219**–**3 430 867)**		**1 280 (988**–**1422)**	**2 094 (1 617**–**2320)**

CI: confidence interval; NA: not available; PM_2.5_: particulate matter with particles ≤ 2.5 µm in diameter; WHO: World Health Organization.

^a^ The estimated attributable proportion is a population-normalized value that reflects the percentage of total all-cause deaths that are attributable to PM_2.5_ exposure. We calculated the attributable proportion as follows: estimated number of attributable cases divided by (population per 100 000 × mortality rate per 100 000).

^b^ Population at risk are individuals 30 years or older.

Note: The 2005 WHO air quality guidelines for annual PM_2.5_ concentration is a maximum target of 10 µg/m^3^,^10^ and for the 2021 guidelines the maximum target concentration is 5 µg/m^3^.^9^

Because the annual concentrations of PM_2.5_ in Australia, Brunei Darussalam and New Zealand are already lower than the 2005 guidelines, these countries would only avoid deaths if they achieved the 2021 guidelines. Australia would avoid 2670 deaths (18 deaths per 100 000 population), Brunei Darussalam 10 deaths (5 deaths per 100 000 population) and New Zealand 180 deaths (7 deaths per 100 000 population). China could avoid the most deaths if achieving either the 2005 guidelines (2 536 156 deaths; 298 deaths per 100 000 population) or 2021 guidelines (2 808 356 deaths; 330 deaths per 100 000 population; Table 2). 

### Economic assessment

If Member States in the Western Pacific Region meet the 2021 guidelines, there would be an estimated annual economic benefit of US$ 4.604 trillion, resulting from the estimated 3.119 million deaths avoided. China could gain the greatest annual economic benefit of US$ 3.830 trillion, followed by Japan (US$ 0.426 trillion) and Republic of Korea (US$ 0.197 trillion; Table 3).

**Table 3:** Estimates of economic benefits of achieving 2021 WHO targets for PM_2.5_ concentrations, Western Pacific Region

Country	Value of a statistical life, in million US$	Total economic benefit, in million US$ (95% CI)	Total economic benefit, US$ per capita (95% CI)
Australia	10.335	27 595 (20 939–30 871)	1 876 (1 423–2 098)
Brunei Darussalam	6.539	65 (52–72)	308 (246–339)
Cambodia	0.184	1 894 (1 459–2 103)	298 (230–331)
China	1.364	3 830 598 (3 016 242–4 208 060)	4 496 (3 540–4 939)
Fiji	0.831	200 (153–224)	504 (385–565)
Japan	6.682	426 245 (324 652–475 885)	4 602 (3 505–5 137)
Kiribati	0.583	12 (9–13)	282 (211–310)
Lao People's Democratic Republic	0.299	1 373 (1 058–1 524)	541 (417–601)
Malaysia	1.819	21 999 (16 823–24 509)	1 516 (1 159–1 689)
Micronesia (Federated States of)	0.612	15 (11–16)	352 (264–382)
Mongolia	0.666	2 745 (2 145–3 026)	2 020 (1 579–2 227)
New Zealand	6.885	1 239 (936–1 384)	450 (340–502)
Papua New Guinea	0.385	10 663 (8 117–11 907)	254 (194–284)
Philippines	0.611	29 916 (22 930–33 296)	713 (547–794)
Republic of Korea	4.723	196 519 (151 476–218 132)	5 739 (4 423–6 370)
Samoa	0.676	25 (19–28)	347 (262–384)
Singapore	8.962	21 778 (16 696–24 242)	5 781 (4 432–6 435)
Solomon Islands	0.330	29 (22–33)	143 (109–159)
Tonga	0.736	18 (14–21)	475 (361–532)
Vanuatu	0.545	27 (20–29)	282 (213–311)
Viet Nam	0.342	31 486 (24 379–34 872)	652 (505–722)
**Total**	**2.577^a^**	**4 604 440 (3 608 157–5 070 247)**	**31 630 (24 345–35 110)**

CI: confidence interval; US$: United States dollars; WHO: World Health Organization.

^a^ Average of all countries.

Note: The 2021 WHO air quality guidelines for annual PM_2.5_ concentration is a maximum of 5 µg/m^3^_._^9^

**Fig. 2:**
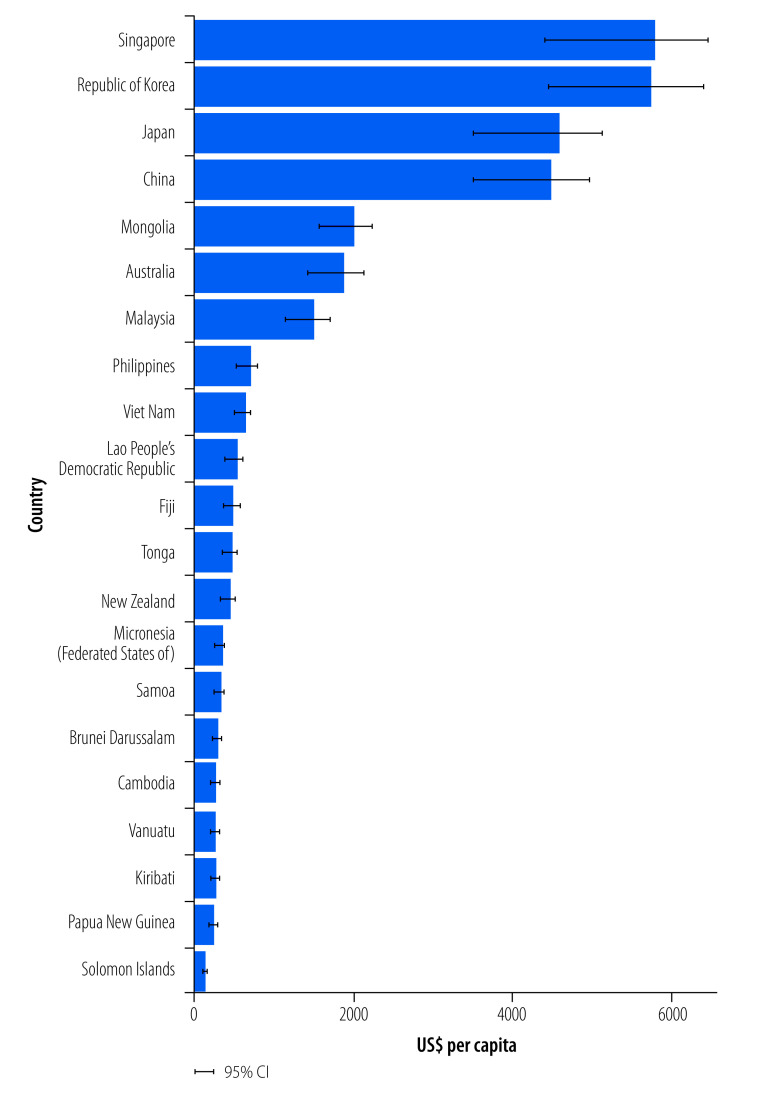
Per capita mortality economic benefit per Member State of the WHO Western Pacific Region related to achieving 2021 WHO air quality guidelines for annual PM_2.5_ concentration

CI: confidence interval; US$: United States dollars; WHO: World Health Organization.

Note: We estimated the economic benefit per capita by multiplying the number of avoidable deaths with the value of a statistical life and divided this result with the 30 years or older population size.

China, Japan, the Republic of Korea and Singapore would gain the largest economic benefit per capita if they achieved the 2021 WHO targets for PM_2.5_ concentrations. The Republic of Korea and Singapore would both gain nearly US$ 6000 per capita per year, while China and Japan would gain approximately US$ 4500 per capita per year. All other countries would gain US$ 2000 or less per person per year, with most countries below US$ 1000 per person (Fig. 2 and Table 3).

## Discussion

In the Western Pacific Region, millions of lives and trillions of dollars could be saved annually by achieving the 2021 WHO air quality guidelines for PM_2.5_. While the number of avoided deaths varied greatly by country, we observed the greatest number, even when adjusting for population size, in China, followed by Mongolia and Viet Nam, which may be attributed to these countries having the highest observed PM_2.5_ concentrations. While some countries with lower PM_2.5_ concentration levels and/or small populations would avoid fewer total deaths (e.g. Australia, Singapore and New Zealand), the higher values of a statistical life in these countries made the associated economic benefits higher. The substantial heterogeneity seen in value of a statistical life estimates across countries may be attributable to differences in income levels, life expectancies, age distributions and social norms regarding risk and death.^20^ According to latest available data, three countries have achieved the 2005 guidelines for the ambient annual PM_2.5_ concentration, while none have achieved the 2021 target. However, it should be noted that the data used in this study were collected in 2016, years before the 2021 target became available. The current evidence base suggests that there is no safe concentration threshold, and improvements to air quality below the current air quality guidelines are expected to bring further benefits.^9^

Our estimated number of avoidable deaths from ambient PM_2.5_ in the Western Pacific Region is approximately three quarters of the 4.2 million deaths worldwide estimated by the 2019 Global Burden of Disease Study.^2^ The economic benefit is approximately equivalent to the 2020 gross domestic product (GDP) of Japan, and one third of China’s 2020 GDP, two of the largest economies in the Region.^21^

For six countries, all small island states, we were unable to identify population and mortality data; thus, we had to exclude these countries from the analysis. Mortality and/or population data may be difficult to obtain for small island states due to resource constraints. This issue raises the important question as to whether countries can also allocate resources for interventions necessary to achieve air quality guidelines, despite the economic benefits. While we did not perform a cost–benefit analysis, studies using such an analysis have shown net benefits for action on air pollution, on their own^22^ or in combination with climate change mitigation actions.^23^ The *25 clean air measures for Asia and the Pacific* report, based on high-quality data and state-of-the-art modelling, suggests that if actions are properly implemented in Asia and the Pacific, it may positively affect the health of 1 billion people at a cost of only 5% of the projected annual GDP increase.^11^ Therefore, countries might be able to achieve the 2021 air quality guidelines without enduring large economic burden to reduce air pollution levels.

We have used a simple example to demonstrate the usefulness of a health impact assessment and monetization of the avoidable deaths. Policy-makers may want to expand on our example through time trends, such as conducting the same health impact assessment for 2005–2009, to compare to our study period of 2015–2019 and demonstrate whether countries are on track towards meeting air quality guidelines. Policy-makers may advance on our methods and concepts by obtaining more granular information to help with air pollution control abatement measures, for example, using gridded global concentration (alongside population) data to estimate differences in health impacts between rural and urban areas, if ground-level monitoring does not yet exist. A recent review of studies within the Asia-Pacific region concluded that methods of assessing exposure (e.g. ground-level monitoring) should be improved.^3^ Nevertheless, it may be useful to assess emissions inventories per country, sector or source, if available (or modelled inventory data if not), to advocate for air pollution mitigation measures targeting specific sectors and sources. For example, policy-makers may refer to the Climate Trace independent greenhouse gas emissions tracking database.^24^

Considering other air pollutants, such as nitrogen dioxide and ozone, may be useful to obtain a more complete estimate of the overall health burden due to air pollution. However, to do such analysis, robust concentration–response functions for these pollutants must be available. Our results could be built upon by performing further analyses using the AirQ+ embedded exposure-response functions for outdoor air pollution, which also consider household air pollution and second-hand smoke exposures. These health impact assessments could consider cause-specific mortality, including chronic obstructive pulmonary disease, lung cancer, stroke and ischaemic heart disease. In AirQ+, these cause-specific mortality rates are based on the 2019 Global Burden of Disease Study.^25^ However, cause-specific mortality rates for Western Pacific Region Member States are not yet available from the UN data source that we used; an alternative non-UN source for these data may be the Global Health Data Exchange.^26^ Policy-makers could also consider country-specific health impact assessments using local rather than global concentration–response functions for all-cause mortality;^27^ however, there may not yet be an adequate number of local studies on outdoor PM_2.5_ pollution and health for conducting a meaningful meta-analysis. While deaths are the main driver of economic benefits because of the high value of a statistical life, investigating years of life lost and value of a statistical life year (i.e. the economic value of illness preceding death, but not death itself) might be useful for having a more complete picture of the economic benefits of achieving air quality guidelines; however, the cost of illness is much lower than the cost of death.^25^

By design, the different counterfactual scenarios in health impact assessments support decision-makers to identify which regulations best meet their economic and industry criteria for future strategic goals. The *25 clean air measures for Asia and the Pacific* report details 25 specific policy actions that can be directly implemented within the Western Pacific Region to decrease PM_2.5_ exposure levels and thus improve public health.^11^ The report groups these policy actions into three different categories, one of which focuses specifically on reducing conventional emissions that lead to PM_2.5_ formation. With the Western Pacific Region Member States expected to grow their economies collectively by approximately 80% by 2030, the report focuses on strengthening compliance from industry and energy-related emission sources (e.g. power plants and transport). For example, the regulation of the transport sector to reduce diesel emissions from heavy vehicles, which is a major source of PM_2.5_, could greatly improve public health. Focusing on emissions controls and air pollution prevention programmes, as the *25 clean air measures for Asia and the Pacific* report states, along with the findings in this study on economic and health impacts, could support policy-makers to make more effective changes in air quality for improved population health.

Our study has some limitations. First, the concentration–response functions are based on published studies mainly from Europe and North America and might not be generalizable to Western Pacific Region Member States. Second, we used PM_2.5_ data averaged for the whole country, and did not distinguish between rural and urban areas. Some cohort studies in the Asia-Pacific region (namely China and Republic of Korea) have suggested different health effects from long-term exposure to PM_2.5_ between rural and urban areas of residence.^28^^,^^29^ Such differences may in part be due to different sources of PM_2.5_ such as greater household air pollution among rural households.^3^ Not being able to consider subregional variability within Member States, due to a lack of data and in-built functionality of AirQ+, hinders us in our study of the differences among attributable deaths within urban versus rural areas of residence. This variability may prove to be important given that some of the main sources of PM_2.5_ come from urban (e.g. commercial, industrial and transport) activities. Future work may create subregional health impact assessments within AirQ+, or perform the health impact assessment outside of AirQ+ using more sophisticated software, such as geographic information systems. Finally, we did not differentiate between gender, however women may experience higher risk than men due to additional PM_2.5_ exposures from household air pollution when performing gender normative roles,^30^ compounding health inequality.^31^

WHO-supported AirQ+ software is an adequate advocacy tool for exercises in risk communication. The United States Environmental Protection Agency has compared their BenMAP tool to AirQ+ and found the results to be comparable.^32^ However, AirQ+ is more user-friendly than BenMAP and thus more likely to be used by policy-makers. AirQ+ users can customize the concentration–response functions available in AirQ+, allowing the user to evaluate cause-specific or source-specific deaths. We annualized 5-year aggregated data, which provides the advantage of smoothing out year-to-year variation in mortality and population data. This advantage is important for countries with smaller populations in which estimated attributable deaths could be sensitive to outliers.

Here we show that implementing effective policy and regulations to reduce PM_2.5_ emissions and exposure by meeting the WHO air quality guidelines could avoid deaths and produce an economic benefit for the Western Pacific Region. Member States may require assistance to better monitor ambient air pollution levels, collect mortality and population data and obtain local GDP-adjusted values of a statistical life to improve decision-making for mitigative actions.

Note: The 2005 WHO air quality guidelines for annual PM_2.5_ concentration is a maximum of 10 µg/m^3^,^10^ and for the 2021 guidelines the maximum concentration is 5 µg/m^3^.^9^ We estimated the number of avoidable deaths using the AirQ+ tool.^17^
